# Supplementary material for: Complexome profiling of the Chlamydomonas psb28 mutant reveals TEF5 as an early PSII assembly factor
Source: Plant Cell. 2025 Mar 17;37(6):koaf055. doi: 10.1093/plcell/koaf055 (PMC12164590; doi:10.1093/plcell/koaf055)
Supplement: koaf055_Supplementary_Data [file koaf055_supplementary_data.zip › psb28_interactive.zip!/plots/intensity/qualityPlots/peptides/PBGD1_Cre16.g663900.t1.2.html]

### Cre16.g663900.t1.2

Trivial name: PBGD1  
SeqCoverage [median]: 10.90  
Mass: 37.37 kDa  
  
 

| Sequence | MissedCleavage | Mass [Da] | wtIntensity | muIntensity | Score |
| --- | --- | --- | --- | --- | --- |
| EDVRDVFISPVAK | 1 | 1473.78 | 273.21 | 0.00 | 33.20 |
| ILNQPLADIGGK | 0 | 1237.70 | 0.00 | 1101.37 | 93.65 |
|  |  |  | 273.2 | 1101.4 |  |
